# Time-resolved MRA using sliding window reconstruction for evaluation of renal arterial anatomy and perfusion

**DOI:** 10.1186/1532-429X-11-S1-P130

**Published:** 2009-01-28

**Authors:** Aoife N Keeling, Ravi K Singh, Cormac Farrelly, Hyun Jeong, Ty A Cashen, John Sheehan, Tim J Carroll, James C Carr

**Affiliations:** Northwestern Memorial Hospital, Chicago, IL USA

**Keywords:** Magnetic Resonance Angiography, Renal Transplant Patient, Renal Perfusion, Deconvolution Method, Gadopentetate Dimeglumine

## Introduction

Time-resolved contrast enhanced magnetic resonance angiography, if fast enough, can not only provide arterial anatomical and pathological detail, but can also follow the first pass of contrast through visceral parenchymal tissue in order to evaluate vascular flow dynamics or perfusion.

## Purpose

To evaluate the feasibility of a new magnetic resonance imaging (MRI) technique that would provide unified anatomic and functional evaluation of the kidneys in a single scan with a single dose of Gadolinium contrast. We hypothesize that dynamic mask-mode subtraction yields perfusion weighted images of the renal parenchyma.

## Methods

A new magnetic resonance angiography (MRA) pulse sequence, with a high frame rate, capable of simultaneous determination of renal arterial anatomy and pathology, and renal perfusion, was developed. Eleven healthy volunteers and one renal transplant patient were recruited to undergo MRI examination using a radial three-dimensional FLASH acquisition with sliding window view-share reconstruction (in-plane spatial resolution was 1.1 × 1.1 mm^2^) on a 1.5 T Siemens MRI scanner. A single dose of Magnevist (gadopentetate dimeglumine, Berlex, Montville, NJ, USA) was administered intravenously. Images were processed with a dynamic mask-mode subtraction which has been shown to be beneficial in artery-vein separation. The raw data was reconstructed offline with a sliding window and a sliding mask subtraction technique generating sequential angiographic images at a rate of 3 frames per second. Perfusion analysis was also performed offline by two experienced diagnostic radiologists, implementing the upslope and deconvolution methods, as well as using the maximum value subtracted image. All reconstructions and analyses were performed using Matlab software. The maximum value subtraction image was compared on a pixel-by-pixel basis to the upslope and deconvolution methods using correlation and Bland-Altman plots.

## Results

The technique produced diagnostic quality angiographic images and perfusion maps in all volunteers and the renal transplant patient. The maximum value subtracted image provided an accurate estimate of renal perfusion as compared to upslope and deconvolution methods using correlation plots (r = 0.98, 0.94; m = 0.96, 0.89) and Bland-Altman plots (mean bias = 1.7%, 2.1%; p < 0.05).

## Conclusion

This technique provides anatomic and functional evaluation of the kidneys with a single scan and single dose of contrast that would currently require multiple scans or examinations that require ionizing radiation. The maximum value subtracted image provides an accurate estimate of renal perfusion compared to the established upslope and deconvolution methods. This technique could be applied clinically to a population of potential renal transplant donors, providing arterial anatomic information for surgical planning as well as pre-transplant functional evaluation.

